# Percutaneous endoscopy in direct real-time observation of choke vessels in rat perforator flap model

**DOI:** 10.1016/j.jpra.2019.01.008

**Published:** 2019-02-10

**Authors:** Xuchang Meng, Zhichao Wang, Xiangwen Xu, Jieyi Ren, Xin Huang, Yihui Gu, Bin Gu, Qingfeng Li, Tao Zan

**Affiliations:** a Department of Plastic and Aesthetic Sugery, First Affliated Hospital of Guangxi Medical University, 6 Shuang Yong Road, Nanning, Guangxi 530021, PR China; b Department of Plastic and Reconstructive Surgery, The Ninth People's Hospital, Shanghai Jiao Tong University School of Medicine, 639 Zhizaoju Road, Shanghai 200011, PR China

**Keywords:** Percutaneous endoscopy, Choke vessel, Visualization, Perforator flap model

## Abstract

**Background:**

Most of the techniques used to investigate choke vessels are indirect. The aim of the present study is to assess the effectiveness of percutaneous endoscopy in direct real-time visualization of choke vessels in rat perforator flap models.

**Methods:**

A classic perforator flap on the rat dorsum was designed (*n* = 12). An additional incision was made to place the percutaneous endoscope. Evans blue dye was injected from the common carotid artery to distinguish choke arteries from veins. Blood perfusion status was assessed using full-field laser perfusion imaging (FLPI) and the oxygen/carbon dioxide levels. Photographs of choke vessels were taken and compared at 1 h, 1 day, 4 days, and 7 days postoperation. The flap survival area was examined on day 7.

**Results:**

The average survival rate of perforator flaps was 70.1 ± 10.8%. The choke arteries but not choke veins were stained blue after injection of Evans blue dye. The choke arteries constricted instantly after surgery, dilated to a maximum diameter on postoperation day 4, and returned to the preoperation status on day 7. The choke veins dilated instantly after the operation, reached their largest diameters on postoperation day 4, and remained dilated on day 7. The behaviors of choke vessels were consistent with the FLPI results and oxygen/carbon dioxide statuses.

**Conclusion:**

Percutaneous endoscopy can provide direct real-time visualization of choke vessels in living rat perforator flap models and enable the identification of choke arteries and veins. This novel technique represents an ideal platform for investigating choke vessels in perforator flap models.

## Introduction

The perforator flap has been the focus of plastic surgery research for decades owing to its flexibility of surgical design and minimal trauma to donor site.^1^, ^2^ However, one major drawback of this surgical technique is the blood supply of perforator flap, which is not always sufficient to meet the needs of massive trauma reconstruction. The existing concepts of perforator flaps were mainly established with morphological studies of flaps conducted by Taylor and colleagues in the 1980s; through careful study, these researchers observed some delicate vessels, which they then named “choke vessels”.^3^ Choke vessels are reduced-caliber vessels that connect adjacently under physiological vascular conditions.^4^ The behaviors of choke vessels influence the survival of perforator flaps. Consequently, further study of choke vessels may provide a theoretical basis for the establishment of cross-angiosome perforator flaps.

Research on choke vessels has been performed for more than 30 years.^3^, ^4^ Initially, elaborate anatomical studies and vascular perfusion methods were used.^5^ These methods enabled the intuitive observation of choke vessels, but the experimental animal had to be killed, and direct observation could be obtained only a single time. New imaging methods such as computed tomography angiography (CTA), magnetic resonance angiography (MRA), and digital subtraction angiography (DSA) have been applied clinically to blood vessels.^6^ However, it is difficult to distinguish choke vessels with small diameters using these techniques. In recent years, some scholars have developed skinfold chambers to observe the choke vessels on the rat's skin flap, but potential high infection rates remain an obstacle to the application of this technique, and a preselected zone might not be an ideal site for choke vessel observation.^7^

In the present study, we utilized percutaneous endoscopy to directly visualize and investigate the choke vessels in a rat skin perforation flap model. The objectives of this study were to assess the usefulness of percutaneous endoscopy in the real-time direct observation of choke vessels and to provide a safe and optimal platform for further research focusing on artery perfusion, venous drainage, and perforator flap survival.

## Methods

### Animals

All study protocols were approved by the Institutional Animal Care and Use Committee of the Ninth People's Hospital of Shanghai Jiao Tong University. Twelve adult male Sprague–Dawley (SD) rats with an average weight of 250–300 g were included. The rats were housed in groups of three on a 12-hour light/dark cycle with food and water available. During the percutaneous endoscopic observations, all animals were anesthetized using an intraperitoneal injection of pentobarbital (50 mg/kg).

### Perforator flap model

**Figure 1:**
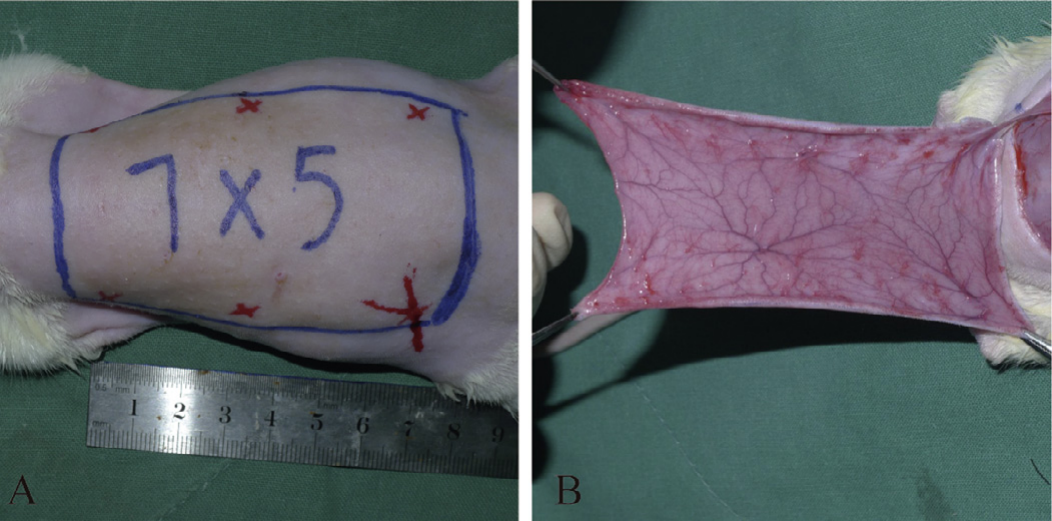
Establishment of the rat dorsal perforator flap model.

The rats were anesthetized with an intraperitoneal injection of sodium pentobarbital at a dose of 60–90 mg/kg, and additional doses were used when necessary during the surgery.^8^ Following standard aseptic techniques, the dorsal hair was removed, and the skin was cleaned with 2% povidone-iodine solution. A caudally based 7 × 5 cm^2^ dorsal perforator flap was raised in each rat through sharp dissection between the iliac crests (Figure 1A). The upper margin was at the caudal tip of the scapula, and the lower margin was 2 cm below the iliac crest. The region contained the thoracodorsal (TD) arteries, the posterior intercostal (PIC) arteries, and the deep circumflex iliac (DCI) arteries of both sides.^5^ The corresponding choke vessels exist between the adjacent angiosomes.^5^ As previously described,^9^ only the left DCI artery was protected, and other arteries were ligated to establish the perforator flap model (Figure 1B). The flap was sutured to the donor site with 4-0 silk sutures.

### Percutaneous endoscopic observation

**Figure 2:**
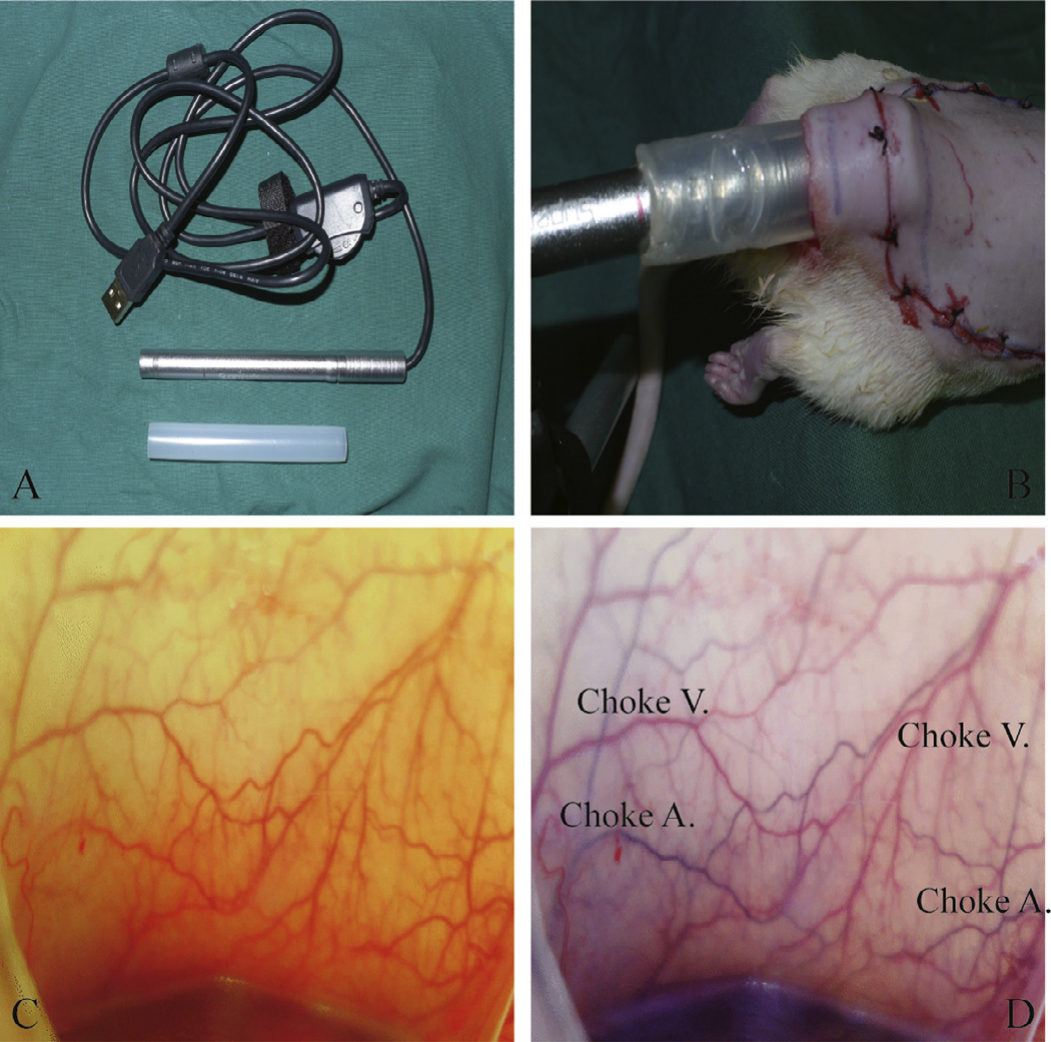
The application of percutaneous endoscopy in the perforator flap rat model.

After the elevation of the skin flap, the anastomotic zone between the left and right iliolumbar arteries was marked on the surface of the perforator flap. The percutaneous endoscopic observation was consistently focused on this target zone. Next, an incision was made 2 cm away from the caudal edge of the flap, and a commercially available percutaneous endoscope (B003+, Shenzhen Supereyes Technology Co. Ltd., Shenzhen, China) was placed through this incision (Figure 2B). Direct real-time observation of the microcirculation in the choke vessels was recorded (Figure 2C). Photographs were taken at 1 h, 1 day, 4 days, and 7 days postoperation. The arteries and veins in the choke vessels were further identified by the injection of Evans blue dye (Sigma, St. Louis, MO) from the common carotid artery (Figure 2C and D: before and after the injection, respectively).

### Full-field laser perfusion imaging (FLPI)

As previously described,^10^ FLPI is a well-recognized method for assessing the temporal changes in the circulation of flaps. FLPI was conducted in a low-resolution/high-speed setting at a display rate of 25 Hz with a camera exposure period of 20 ms and a time constant of 0.3 s. Contrast images were produced using a scaled color-coded Live Flux image (red and blue indicate high and low perfusion, respectively) that correlated with the blood flow velocity in the prefabricated flaps.

### Oxygen/carbon dioxide measurements

Percutaneous oxygen/carbon dioxide levels were measured using a TCM4 (Radiometer Medical A/S, Copenhagen, Denmark) in the same target zone as that of the percutaneous endoscopic observations. The measurements were conducted after the endoscopic observations when the rats were under anesthesia.

### Flap survival analysis

On postprocedure day 7, flap survival was estimated by measuring the sizes of the viable and ischemic areas using a transparent sheet. Digital images of the flap were recorded on a computer, and the survival was calculated as a percentage of the viable area of the whole flap. The survival rate was calculated as the viable area/whole area. The viable tissue was demarcated grossly in a blinded fashion on the basis of texture, color, and appearance by two different observers. The sheet was then scanned, and the survival area was calculated in square centimeters using Image Pro Plus software (version 6.0).

### Statistical analysis

All values are presented as the mean ± standard deviation (SD). Statistical significance was determined using Student's t-test with an alpha of 0.05. Statistical analyses were performed using SPSS version 16.0 (SPSS Inc., Chicago, USA).

## Results

### Establishment of perforator flap rat model

**Figure 3:**
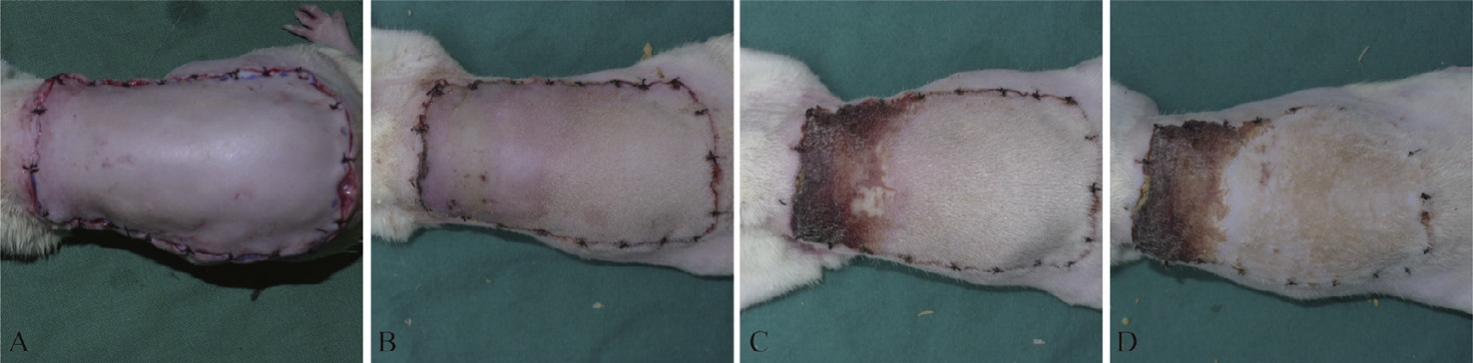
Gross observation of the rat dorsal perforator flap model.

Instant assessments revealed that the operations were successful and without active bleeding, apparent swelling, or other complications (Figure 3A). All rats survived the operations and woke up after anesthesia. At postoperation day 2, the distal part of the flaps became bruised and swollen and expanded in size (Figure 3B). During postoperation days 3–4, the swelling of flaps gradually subsided, and the distal parts of the flaps turned brown, acquired a hard texture, and exhibited loss of hair (Figure 3C). These changes in the distal part indicated flap necrosis. From postoperation day 5, the size of distal necrosis became stable with clear boundaries. The final survival area and corresponding survival rate of the dorsal perforator flaps were assessed on postoperation day 7. The average survival rate of the perforator flaps was 70.1 ± 10.8% (Figure 3D), which is consistent with the results of previous studies.^11^

### Observation of choke vessels using percutaneous endoscopy

**Figure 4:**
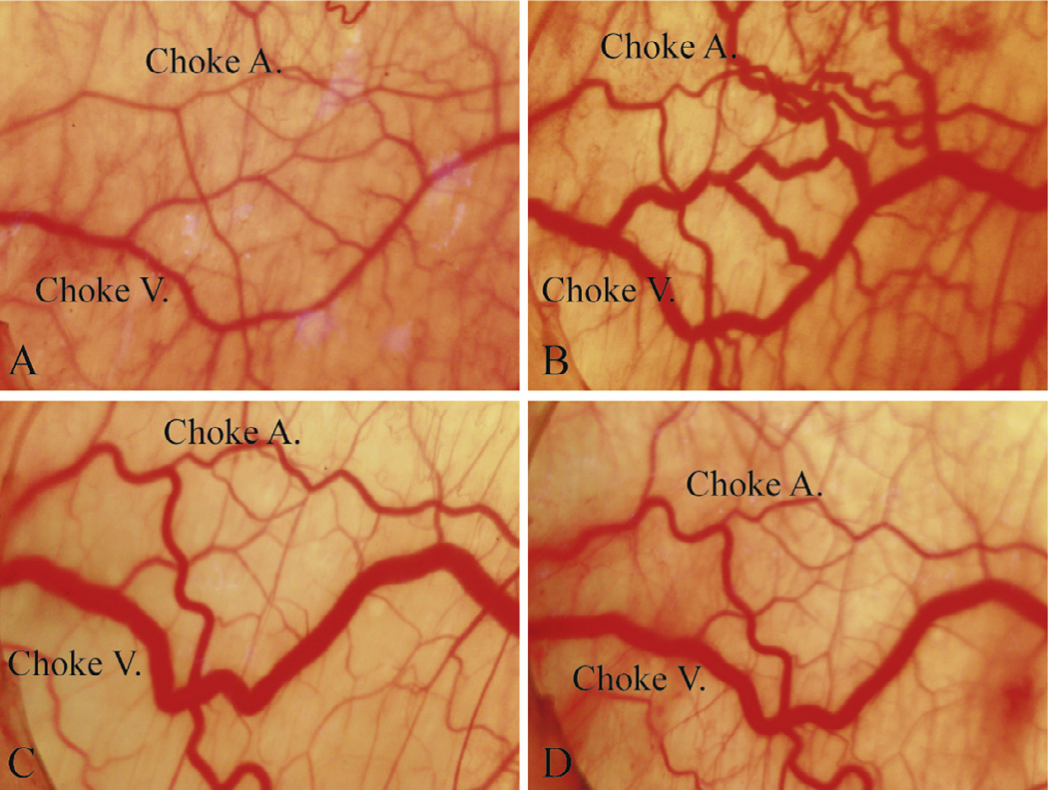
The rats’ choke vessels in the perforator flap observed by percutaneous endoscopy.

Instant observations of the anastomotic zone between the left and right DCI arteries in the perforator flap revealed both the perforator arteries and veins (Figure 4A). The perforator arteries had smaller diameters and fewer branches than the perforator veins. The choke veins connecting the perforator veins had greater diameters than the choke arteries.

At one-hour post operation, the choke arteries constricted, whereas the choke veins began to dilate and were filled with blood (Figure 4A). On postoperation day 1, both the choke arteries and veins had dilated to significantly larger diameters and exhibited tortuous features, which indicated increased blood flow to the perforator flap from the left iliolumbar arteries (Figure 4B). On postoperation day 4, the maximum diameters of the choke arteries were reached, as was the peak tortuosity. However, the diameters of the choke veins began to decrease (Figure 4C). On postoperation day 7, the extent of dilation and the degree of tortuosity of the choke vessels decreased but remained greater than the original levels (Figure 4D). During the whole process, no apparent neovascularization was detected.

### Identification of the choke arteries and veins

Following a bolus injection of 1 ml of Evans blue dye from the common carotid artery toward the aorta, the whole process of the microcirculation of the choke vessels was video recorded (Online Supplementary file). Six seconds after injection, the choke arteries were stained blue and were distinguishable from the choke veins. The choke arteries were stained blue first after the injection, and then, the choke veins drained the blue dye away. The blue flow velocity was measured from the recorded video and compared between the different time points. A similar technique was reported previously by injection into the lateral tail vein.^7^

### Correlation of the choke vessels’ behaviors between the FLPI and the oxygen/carbon dioxide levels

**Figure 5:**
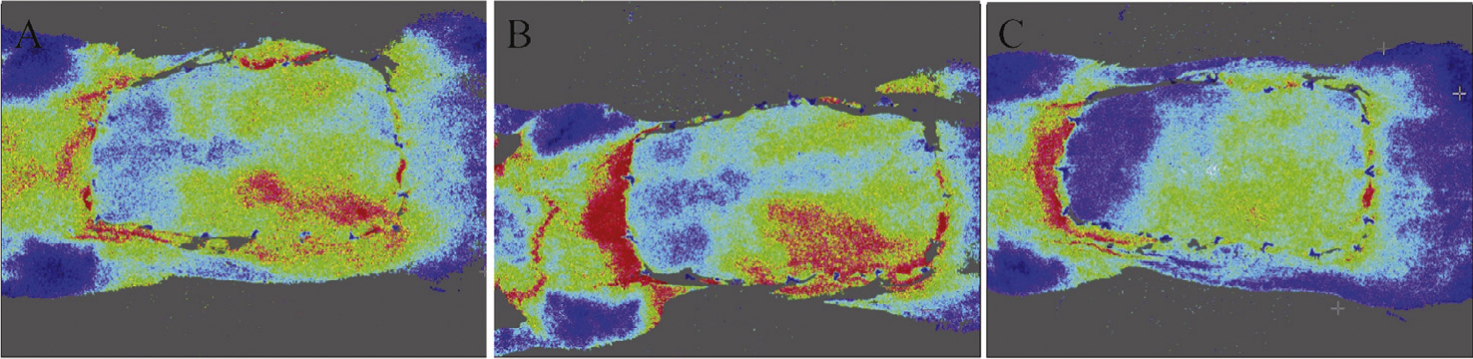
Blood perfusion measured using FLPI in the rat perforator flap model.

The real-time profusion statuses of the perforator flaps were further assessed using FLPI at postoperation days 1, 4, and 7 (Figure 5A–C). The perfusion statuses of the choke zones were significantly correlated with the behaviors of the choke vessels. The maximum blood perfusion in the choke zone was observed on day 4 when the largest dilations of the diameters of the choke vessels were detected under percutaneous endoscopy. The lowest oxygen and highest carbon dioxide levels were detected immediately after surgery, which correlated with the constriction of the choke arteries and blood stasis in the choke veins observed by percutaneous endoscopy (Table 1).

**Table 1:** Percutaneous oxygen/carbon dioxide levels in target choke zone of perforator flap rat model.

Percutaneous	Post-operation			
Measurements	Hour 1	Day 1	Day 4	Day 7
Oxygen	66	46.9	53.2	44.7
Carbon Dioxide	19.2	34.8	24.3	27.8

Note: Oxygen/carbon dioxide were measured in units of PaO_2_ and PaCO_2_.

## Discussion

Because perforator flaps can overcome the shapes and sizes of natural angiosomes through dilating anastomotic choke vessels, they have become well-accepted and useful tools in reconstructive surgeries, especially for large soft-tissue defects.^12^, ^13^ Although various techniques such as infrared thermography have been developed to detect the blood perfusion statuses of perforator flaps, it remains difficult to visualize the statuses of blood perfusion linking different angiosomes. Therefore, it is vital to develop a convenient tool to visualize choke vessels’ behaviors and to establish a platform to investigate the underlying mechanisms.

Endoscopy is a revolutionary technology in the field of surgery. As the first endoscopy was applied to observe a urethral tract in 1805, the technology has been rapidly developed and applied to observe structures and treat diseases in different cavities and tracts in the human body and in animal models.^14^ The most common endoscopy used in clinical practice is laparoscopy and gastrointestinal endoscopy, which aids in diagnosis or therapeutic interventions with a few small cuts in the abdomen or through the digestive tract.^15^ However, this study is the first report of the application of percutaneous endoscopy for the direct observation of choke vessels in a rat perforator flap model that was enabled by the establishment of an artificial cavity under the flap.

Previously, direct observations of choke vessels could only be achieved after sacrificing the model animal.^5^ Our results demonstrated that percutaneous endoscopy can provide continuous real-time monitoring of living animal models. The dilating diameters and increased blood flow in the choke vessels connecting the different angiosomes could be directly assessed. These morphological changes in the choke vessels were consistent with the results from FLPI and percutaneous oxygen/carbon dioxide level experiments. Furthermore, by injecting Evans blue dye, the choke arteries and choke veins could be identified. This technique provides an ideal and precise platform for independently investigating the physiological behavior or drug reactions of choke arteries and veins, which cannot be achieved with previously employed indirect blood perfusion evaluation methods. A similar technique was reported by Zhuang et al who used the lateral tail vein.^7^ However, the Evans blue dye had been diluted by the time it reached the choke arteries, and therefore, the contrast was significantly lower than that achieved using the common carotid artery. Additionally, the required dose of Evans blue dye was low, and the duration of staining was short when the carotid artery was used.

The first blood vessel visualization method was developed by Heinz et al in 1979 to observe skin blood vessels in rats.^16^ With this technique, a skin window was created in living animals. However, the skin window was susceptible to infection and had negative influences on the physiological behaviors of the experimental animals.^7^ Percutaneous endoscopy has the following significant advantages over skin windows: (1) The influence on the experimental animals is minimal, and the complication rates, including infection, are low. Without these potentially influential factors associated with the observation technique, the results can reflect the physiological condition or the influences of indentations. (2) Percutaneous endoscopy can increase the resolution and thus be used to identify tiny blood vessels by magnification. Therefore, small animal models such as rats can be used for the direct observation of choke vessels. (3) Continuous monitoring of the choke vessels’ changes in the same rat can decrease intersubject heterogeneity to a minimal level. Such continuous monitoring could potentially be applied in a clinical setting to provide personalized practices and interventions for patients.

Percutaneous endoscopy also has several limitations: (1) It is relatively difficult to achieve the same observation zone every time due to changes in the position and rotation of the camera, changes in the body statuses of the rats, etc. Therefore, comparisons of pictures and the diameters of choke vessels are relatively subjective. (2) The observation zone is a limited area of the whole perforator flap and thus might not be able to reflect the blood perfusion of the entire flap. Targeting different zones using percutaneous endoscopy could be a potential solution for this limitation but that would require multiple camera adjustments, which would be sophisticated and time-consuming. (3) The wound for the insertion of the percutaneous endoscope represents a persistent potential risk of infection, especially if multiple observations are to be made over a long period.

## Conclusions

Percutaneous endoscopy can provide direct real-time visualizations of choke vessels in living rat perforator flap models that enable the identification of choke arteries and choke veins. This novel technique represents an ideal platform for further investigating the influence of choke vessels in perforator flap models in terms of arterial perfusion, venous drainage, and flap survival.

## Appendix Supplementary materials

Image, video 1

Image, application 2
