# Supplementary material for: Percutaneous endoscopy in direct real-time observation of choke vessels in rat perforator flap model
Source: JPRAS Open. 2019 Feb 10;20:27–34. doi: 10.1016/j.jpra.2019.01.008 (PMC7061670; doi:10.1016/j.jpra.2019.01.008)
Supplement: Supplementary file 2 [file mmc2.docx]

**Table S1. Percutaneous Oxygen/Carbon Dioxide Levels in target choke zone of the perforator flap rat model.**

| **Percutaneous** | **Postoperation** | | | |
| --- | --- | --- | --- | --- |
| **Measurements** | **Hour 1** | **Day 1** | **Day 4** | **Day 7** |
| **Oxygen** | 66 | 46.9 | 53.2 | 44.7 |
| **Carbon Dioxide** | 19.2 | 34.8 | 24.3 | 27.8 |

Note: Oxygen and Carbon Dioxide were measured in units of PaO_2_ and PaCO_2_.
